# A Deep Learning-Based Automated CT Segmentation of Prostate Cancer Anatomy for Radiation Therapy Planning-A Retrospective Multicenter Study

**DOI:** 10.3390/diagnostics10110959

**Published:** 2020-11-17

**Authors:** Timo Kiljunen, Saad Akram, Jarkko Niemelä, Eliisa Löyttyniemi, Jan Seppälä, Janne Heikkilä, Kristiina Vuolukka, Okko-Sakari Kääriäinen, Vesa-Pekka Heikkilä, Kaisa Lehtiö, Juha Nikkinen, Eduard Gershkevitsh, Anni Borkvel, Merve Adamson, Daniil Zolotuhhin, Kati Kolk, Eric Pei Ping Pang, Jeffrey Kit Loong Tuan, Zubin Master, Melvin Lee Kiang Chua, Timo Joensuu, Juha Kononen, Mikko Myllykangas, Maigo Riener, Miia Mokka, Jani Keyriläinen

**Affiliations:** 1 Docrates Cancer Center, Saukonpaadenranta 2, FI-00180 Helsinki, Finland; timo.joensuu@docrates.com (T.J.); juha.kononen@docrates.com (J.K.); mikko.myllykangas@docrates.com (M.M.); maigo.riener@kymsote.fi (M.R.); 2 MVision Ai, c/o Terkko Health hub, Haartmaninkatu 4, FI-00290 Helsinki, Finland; saad.akram@mvision.ai (S.A.); jarkko.niemela@mvision.ai (J.N.); 3 Department of Biostatistics, University of Turku, Kiinamyllynkatu 10, FI-20014 Turku, Finland; eliisa.loyttyniemi@utu.fi; 4 Kuopio University Hospital, Center of Oncology, Kelkkailijantie 7, FI-70210 Kuopio, Finland; jan.seppala@kuh.fi (J.S.); janne.heikkila@kuh.fi (J.H.); kristiina.vuolukka@kuh.fi(K.V.); okko.kaariainen@kuh.fi (O.-S.K.); 5 Oulu University Hospital, Department of Oncology and Radiotherapy, Kajaanintie 50, FI-90220 Oulu, Finland; vesa-pekka.heikkila@ppshp.fi (V.-P.H.); kaisa.lehtio@ppshp.fi (K.L.); juha.nikkinen@ppshp.fi (J.N.); 6 University of Oulu, Research Unit of Medical Imaging, Physics and Technology, Aapistie 5 A, FI-90220 Oulu, Finland; 7 North Estonia Medical Centre, J. Sütiste tee 19, 13419 Tallinn, Estonia; Eduard.Gerskevits@regionaalhaigla.ee (E.G.); anni.borkvel@regionaalhaigla.ee (A.B.); merve.adamson@regionaalhaigla.ee (M.A.); Daniil.Zolotuhhin@regionaalhaigla.ee (D.Z.); kati.kolk@regionaalhaigla.ee (K.K.); 8 National Cancer Centre Singapore, Division of Radiation Oncology, 11 Hospital Crescent, Singapore 169610, Singapore; eric.pang.p.p@nccs.com.sg (E.P.P.P); jeffrey.tuan.k.l@singhealth.com.sg (J.K.L.T); zubin.master@nccs.com.sg (Z.M.); melvin.chua.l.k@singhealth.com.sg (M.L.K.C); 9 Oncology Academic Programme, Duke-NUS Medical School, Singapore 169857, Singapore; 10 National Cancer Centre Singapore, Division of Medical Sciences, Singapore 169610, Singapore; 11 Turku University Hospital, Department of Oncology and Radiotherapy, Hämeentie 11, FI-20521 Turku, Finland; miia.mokka@tyks.fi (M.M.); jani.keyrilainen@tyks.fi (J.K.); 12 Turku University Hospital, Department of Medical Physics, Hämeentie 11, FI-20521 Turku, Finland

**Keywords:** deep learning, prostate cancer, radiation therapy, autosegmentation, treatment planning

## Abstract

A commercial deep learning (DL)-based automated segmentation tool (AST) for computed tomography (CT) is evaluated for accuracy and efficiency gain within prostate cancer patients. Thirty patients from six clinics were reviewed with manual- (MC), automated- (AC) and automated and edited (AEC) contouring methods. In the AEC group, created contours (prostate, seminal vesicles, bladder, rectum, femoral heads and penile bulb) were edited, whereas the MC group included empty datasets for MC. In one clinic, lymph node CTV delineations were evaluated for interobserver variability. Compared to MC, the mean time saved using the AST was 12 min for the whole data set (46%) and 12 min for the lymph node CTV (60%), respectively. The delineation consistency between MC and AEC groups according to the Dice similarity coefficient (DSC) improved from 0.78 to 0.94 for the whole data set and from 0.76 to 0.91 for the lymph nodes. The mean DSCs between MC and AC for all six clinics were 0.82 for prostate, 0.72 for seminal vesicles, 0.93 for bladder, 0.84 for rectum, 0.69 for femoral heads and 0.51 for penile bulb. This study proves that using a general DL-based AST for CT images saves time and improves consistency.

## 1. Introduction

Prostate cancer is the most common cancer type among the western male population. Radiation therapy (RT) remains a cornerstone in the curative treatment chain with surgery and hormonal therapies [1,2]. Almost 1.3 million new prostate cancer cases were diagnosed worldwide in 2018, and the number is estimated to increase by 35% in 10 years and even up to 80% by 2040 [1]. Access to RT is geographically unequal, with regions in poverty having longer waiting times and less access to lifesaving RT globally and locally [1,3]. As the number of cancer patients treated with ionizing radiation increases, healthcare services have to manage limited financial and human resources, indicating a need for automated solutions and tools.

High geometric and dosimetric accuracy are critical for successful RT. Wide use of image- and surface-guided radiation therapy (IGRT, SGRT) and target tracking methods ensure accurate treatment targeting and reproducibility of patient positioning. Correspondingly, recent increases in using volumetric- or intensity-modulated radiation therapy (VMAT, IMRT) and stereotactic body radiation therapy (SBRT) have enabled us to shape the dose distributions much more accurately to match the treatment target and minimize the dose to organs at risk (OAR) [4]. However, the sharp dose gradients created by these techniques can be hazardous if the delineation of structures is inaccurate [5]. For prostate cancer, the challenges concerning inter- and intraobserver variation in target delineation have been well known for decades [6,7,8]; however, it remains the weakest link in the accuracy of RT [9].

To save clinicians’ resources, automated segmentation tools (AST) for radiation therapy planning (RTP) have been developed, ranging from adaptive thresholding to atlas-based techniques [10]. However, using such automated techniques has been limited due to the complexity of the anatomy and poor contrasts in the pelvic region in computed tomography (CT) images. In practice, the accuracy and speed of the delineation have not been good enough to ease clinicians’ work burden [11]. More recently, the focus on autosegmentation has shifted to artificial intelligence (AI)-based methods [12,13,14]. AI is a branch of computer science that attempts to emulate human-like intelligence in machines through computer software and algorithms without direct human stimuli. Deep learning (DL) is the latest form of AI concerned with artificial neural network (ANN) algorithms that mimic the human nervous system [12,15]. Each “neuron” receives an input and creates an output in successive layers, capable of weighing the importance of the passing information. DL uses multiple levels of abstraction in its process to learn connections and correlations when provided with a database. It typically requires large amounts of training data and computational resources. A trained DL model generates results accurately and quickly with minimal user interaction, and the output results directly from the learned features from the problem distribution. For the delineation of structures of interest in RTP of prostate cancer, the problem distribution is a medical imaging dataset with segmented contours [16,17,18,19].

In this study, MVision Delineation from MVision AI Oy (Helsinki, Finland), a fully automated commercially available 3D DL model, was evaluated for accuracy and efficiency gain for CT-based autocontouring for prostate cancer RTP. Conventional evaluation metrics were used to evaluate delineation accuracy between the predictions of the model and the ground truths of clinicians’ contouring.

## 2. Materials and Methods

### 2.1. DL Delineation Model

The DL model employed is a convolutional neural network based on an encoder–decoder architecture, similar to 3D U-Net [20] and with more recent residual blocks [21]. To train the DL model, ca. 900 prostate cancer patients’ planning CT image sets from three clinics were collected. Clinical contours with large outliers were excluded from the data. The data used for training the model included 876 samples for bladder, 848 for rectum, 738 for prostate, 416 for penile bulb, 222 for seminal vesicles, 212 for lymph nodes and 127 and 128 for left and right femur heads.

The same pretrained general DL-based AST model was used at all clinics in the study. It is a cloud-based system that uses pseudonymized DICOM data in the autocontouring process. Once it completes the delineations, it sends them automatically back to the clinic’s RTP system for clinicians to review, edit, and approve the results.

### 2.2. Study Patients

This is a retrospective institutional review board- and The Finnish Institute for Health and Welfare (Terveyden ja hyvinvoinnin laitos (THL) Helsinki, Finland) approved multicenter study. All procedures performed in the study were in accordance with the ethical standards of the institutional and/or national research committee and with the Declaration of Helsinki by the World Medical Association and its later amendments or comparable ethical standards.

Thirty prostate cancer patients were randomly selected from six clinics’ databases, concluding five patients per clinic with RTP CT images and corresponding segmentations. One clinic from Estonia (North Estonia Medical Centre, Tallinn), four clinics from Finland (Docrates Cancer Center, Kuopio University Hospital, Oulu University Hospital, Turku University Hospital) and one clinic from Singapore (National Cancer Centre Singapore) participated in the study. The selection criteria for the patients were: RTP for prostate cancer, no prostatectomy and no femoral implants. Scans were not used in the training set to create the DL model. Two GE (Lightspeed RT16), two Siemens (Sensation Open and Definition) and two Toshiba (Aquilion LB) scanners were used for CT imaging of the patients.

For a blind evaluation, the same patient scans were duplicated and anonymized to manual contouring (MC) and automated and edited contouring (AEC) groups then sent back to the clinics for delineation. Automated contouring (AC) without editions were also used for the comparison. All the regions of interest (ROI) for the AEC group were edited from the autosegmentations to reach clinically acceptable contours, whereas the MC group included empty datasets for manual contouring. Six clinics produced seven ROIs including prostate, seminal vesicles, bladder, rectum, femoral heads and penile bulb. The lymph nodes were produced at one clinic.

### 2.3. Contouring

Up to three specialists completed the delineations in each clinic except one, where four oncologists delineated the same 5 patients’ datasets (20 scans altogether) so the interobserver variability could be assessed within the clinic. In this clinic, lymph nodes’ CTV delineations were also evaluated. The analyzed dataset therefore consisted of 30 patients and 45 scans. The OARs were contoured according to each clinic’s clinical protocol either by an oncologist, dosimetrist, radiotherapy technologist or physicist. Contouring was completed in two phases. First, the MC was done by measuring the total time taken for the task per patient. All semiautomatic segmentation tools (such as deformable brush, autothreshold, autopilot, etc.), which were clinically used, could be used for contouring. An RTP system Eclipse (v15.6, Varian Medical Systems Finland Oy, Helsinki, Finland) was used at the Docrates Cancer Center, National Cancer Centre Singapore, Oulu University Hospital and Turku University Hospital for the MC or semiautomatic contouring. Elekta Monaco (v5.11, Elekta Instrument AB, Stockholm, Sweden) was used at the North Estonia Medical Centre and MIM Software (v6.9, MIM Software Inc., Cleveland, OH, USA) at Kuopio University Hospital. A minimum interval of one week was introduced after the MC to minimize possible learning and memory trace of individual patient contouring. Secondly, the same specialists evaluated the autocontouring results, edited the contours until clinically acceptable and measured the total time spent per patient. By the end of phase two, three groups of contours were collected for analysis:

1. An MC group contoured in the study into an empty dataset to meet clinical acceptance.
2. An AC group that included the nonedited automatically created structure set.
3. An AEC group that included the automatically created structures with expert modifications to meet clinical acceptance.

### 2.4. Contouring Analysis

Efficiency gain using the AST was measured by comparing the total contouring times per patient of the MC method and AEC method to reach clinically acceptable contours. Accuracy of the AST was measured by comparing the AC delineation with all MC delineations with the Dice similarity coefficient (DSC), surface Dice similarity coefficient (S-DSC), 95% Hausdorff distance (HD95) and difference in absolute and relative volume (Diff_mm3 and Diff_%). DSC is the most common metric used to compare binary segmentation masks. It is the ratio of the overlap between two masks normalized by their size, and its value ranges between 0 (no overlap) and 1 (perfect overlap). S-DSC [22] measures the proportion of the surfaces of two masks that lie within a given tolerance (in mm) from each other. We used a fixed tolerance of 2.5 mm for all structures. HD is the maximum distance between the surfaces of two masks. Since HD is very sensitive to outliers, we used HD95, which is the 95th percentile of the distances between the surfaces of the masks. Diff_mm3 is the absolute volume difference between the two masks in mm3 and Diff_% is the relative volume difference between the two masks. The similarity metrics were calculated by an in-house developed (Python) code.

Interobserver consistency using the MC method and AEC method was measured in two ROI groups: first, by comparing the produced lymph node segmentations and second, by comparing five ROIs, including the bladder, prostate, penile bulb, rectum and seminal vesicles, by four oncologists on five patients’ CT scans (the same scans for each oncologist) with both methods. Interobserver consistency was determined by calculating the DSCs of each oncologist compared with the other three oncologists.

## 3. Results

**Figure 1:**
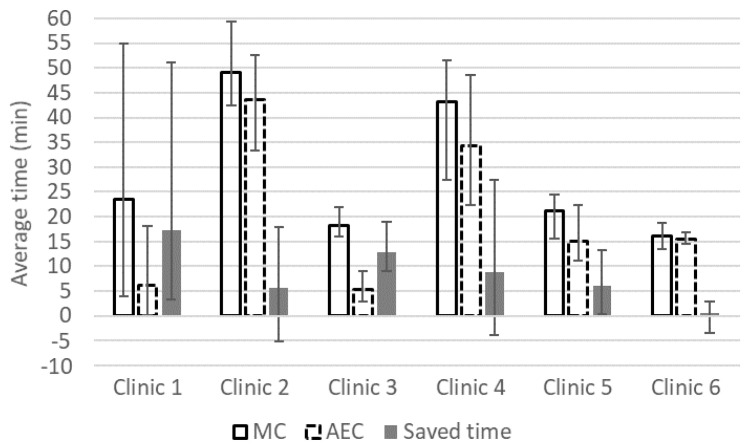
Average manual contouring time (MC bar), automated and edited contouring time (automated and edited contouring (AEC) with dashed line), time saved (filled bar) and range (error bars) in minutes for prostate, bladder, seminal vesicles, rectum, left and right femoral heads and penile bulb.

The efficiency gain in ROI segmentation time using the AST is shown in Figure 1. In all clinics, the average absolute and relative time saved ranged from 0.6 to 17 min and from 1.5% to 70.9%, respectively. Over the expert observers, the absolute and relative time saved ranged from −5 to 51 min and from −25.8% to 96.2%, respectively. The overall average relative time saved for 13 experts contouring 30 patients and 45 scans was 45.8% using the AST. The overall mean absolute MC and AEC delineation times were 27 and 15 min, respectively. For the lymph node delineation, the mean delineation time between four oncologists decreased by 60% from 20 (MC) to 8 min (AEC).

The overall performance of AST is reported in Table 1 in terms of similarity metrics between AC and MC. As seen for the relative volume difference of femoral heads, the results are compromised due to the style differences between the style of the AST and the clinical practice.

**Figure 2:**
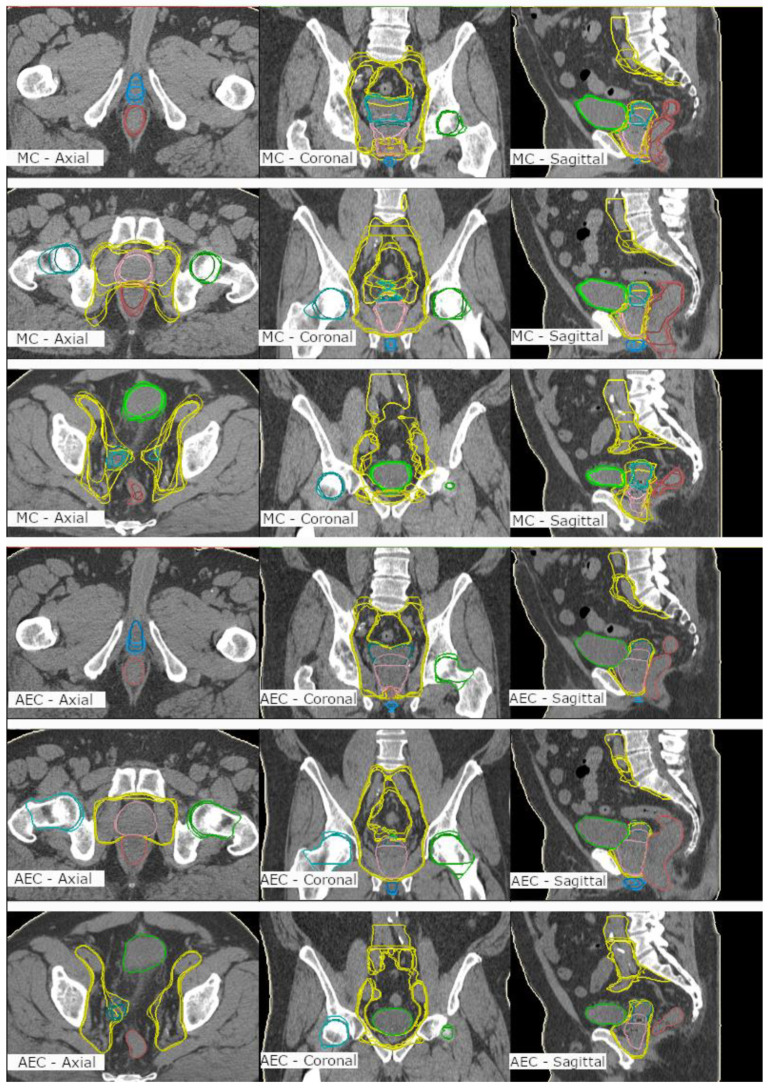
Manual contouring (MC) (**upper 9 images**) and automated and edited contouring (AEC) (**lower 9 images**) of prostate (pink), seminal vesicles (turquoise), lymph nodes (yellow), bladder (light green), rectum (brown), femoral heads (green&light turquoise) and penile bulb (blue) by four oncologists on one patient’s computed tomography scan. Left column: axial plane, middle column: coronal plane; right column: sagittal plane.

The interobserver consistency was studied between the MC and AEC methods by determining the similarity metrics between four experienced oncologists in Clinic 1. The quantitative results are shown in Table 2 for all organs as a five-patients average DSC. For the lymph nodes, the AEC produced higher interobserver consistency between four oncologists when compared with the MC with overall average DSCs of 0.91 ± 0.04 and 0.76 ± 0.04, respectively. For other ROIs, this was consistently evident as well, with overall average DSCs of 0.94 ± 0.06 and 0.78 ± 0.05. The qualitative results may be seen in Figure 2 for all ROIs. By inspecting the resulting ROI segmentations by the four oncologists in Figure 2, one may note better interobserver consistency produced by the AEC than that of the MC, especially for large and challenging ROI, such as the lymph nodes, as confirmed in Table 2.

## 4. Discussion

This study emphasizes that DL-based AST streamlines the workload in clinics and decreases the delineation variability between specialists in clinics. The mean time saved was 12 min or 46% for the full set of prostate RTP contours for six clinics and 12 min or 60% when the regional lymph nodes were also included for RTP at one clinic.

In earlier publications, study groups performed similar studies with 5–20 patients [23,24,25,26,27,28]. As we did not have previous data to evaluate a sufficient sample size for the study using power calculation, we used a greater sample size of 30 patients or 45 scans. Langmack et al. [27] presented an equal delineation time using an atlas-based system for prostate RTP. The mean delineation times for manual and automated segmentations were 27 and 16 min, respectively. However, the atlas-based delineation failed in the delineation of seminal vesicles (DSC of 0.57–0.58) and was only moderate for rectum (DSC of 0.76–0.79), which both are critical organs for the RTP of prostate cancer. In a multicenter study, delineation variation is usually larger than in a more focused or single-center study. Still, our DCSs were consistently higher compared with Langmack’s results, except for femoral heads, where the contour style in the trained model was different when compared with individual clinics. Sjöberg et al. [28] evaluated atlas-based pelvic lymph node delineation and found 23–35% (4–6 min) of mean time saved per patient, which was less than half of the time saved compared with our study for the lymph nodes with 49–75% (10–16 min) of time saved.

Balagopal et al. [16] presented a similar DSC for the DL-based autosegmentation of bladder (0.95) and rectum (0.84). Their DSCs were better for prostate (0.90) and femoral heads (0.95–0.96), but they used only one observer and one CT device as a reference, which would have biased the results if compared with a multicenter study. Their model did not delineate seminal vesicles or penile bulb. Magnetic resonance imaging (MRI) is increasingly used in the RTP of prostate cancer to reduce the uncertainty of target volume delineation. Very recently, Kuisma et al. [29] presented results of a commercial-model-based AST for MRI. In their single-center and single-observer study, the DSCs were similar as in our multicenter CT-based study for prostate (0.82 vs. 0.84), bladder (0.93 vs. 0.92) and rectum (0.84 vs. 0.86). Our model was superior for seminal vesicles (0.72 vs. 0.56) but inferior for penile bulb (0.51 vs. 0.69). MRI has a better soft-tissue contrast compared with CT, which might partly explain the better penile bulb DSCs in MRI. In addition, DSC is strongly affected by the size of the structure, which partly explains poor values for smaller structures, such as the penile bulb.

We added different similarity metrics in Table 1 to make it easier in the future for the research community to compare the similarity metrics between large and small ROIs. The S-DSC measures the segmentation accuracy in relation to the contour lines overlapping rather than the overlapping of the volumes, where the volumes of the ROIs will have a large impact on the DSC, contrary to S-DSC. Examples of this are the DSC of 0.69 and S-DSC of 0.22 for femurs and the DSC of 0.51 and S-DSC of 0.33 for penile bulb. The DSC score decreases more with smaller-volume penile bulb compared with larger-volume femurs, although the contour line overlap is larger for penile bulb than for femurs, as shown by S-DSC. The average relative volume difference between the MC and AC methods for penile bulb and femurs were 6.8% and 34.2%, respectively.

Delineation consistency between the clinics was not possible to evaluate since each clinic only delineated their own patients. However, the interobserver variation was evaluated at one clinic between four experienced oncologists. The consistency increased with the AEC method, as the DSCs for the lymph nodes were 0.76 and 0.91 for the MC and AEC, respectively. The increased consistency is also qualitatively visible in Figure 2, where MC and AEC are presented at three planes. The same was evident for the other organs as well, with overall average DSCs of 0.78 ± 0.05 and 0.94 ± 0.06 for the MC and AEC methods, respectively. The improved consistency will have a great impact in large hospitals with several specialists to standardize treatment or to assist optimal delineation for physicians in training.

**Figure 3:**
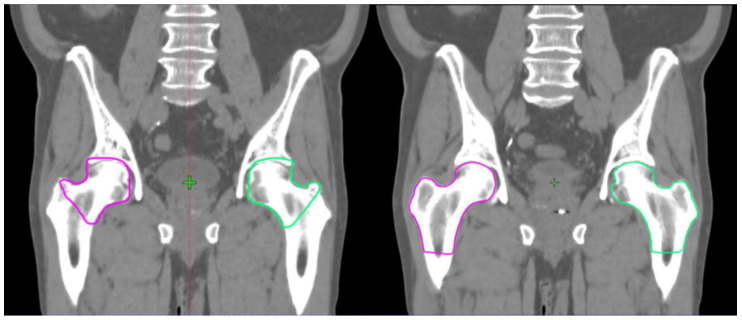
On the **left**, the femoral heads drawn by the automated segmentation tool (AST). On the **right**, the femoral heads drawn by an oncologist at Clinic 6.

In our study, there was a large time-saved variation between the clinics. Clinics 1 and 3 saved 70–71% in the delineation time, while Clinic 6 saved only 1.5% on average. However, time saving was larger when the femurs were excluded from the analysis. For femoral heads, there was a large difference between how the AST was trained and the contouring style used in two clinics (Figure 3). This style difference had an obvious negative impact on the efficiency gain. We compared the change in efficiency gain when femoral heads were excluded from the time save analysis. Absolute and relative time saved increased from 5.6 to 8.1 min (from 10.7% to 18.4%) at Clinic 2 and from 0.5 to 4.6 min (from 1.5% to 31.3%) at Clinic 6.

Langmack et al. found that the DSC should be larger than 0.65 to save time using AST and time saving increases linearly with the DSC [27]. The mean DSC per patient in all clinics was higher than 0.60 and only in two scans below 0.65, indicating there should have been reasonable time saved in all clinics. In this study, the clinics were asked to edit the AC group such that the contours would have been clinically feasible for RTP. However, some clinics used a lot of time to edit the femoral heads, with the lowest DSC of 0.38, which were not autosegmented according to their clinical protocol. This deteriorated the total delineation time but did not have a clinical impact on the RTP. On both occasions, the femoral heads’ doses would have been neglected (V50Gy << 5%, the radiation therapy oncology group (RTOG) consensus) [30]. The current version of the model follows the ESTRO ACROP consensus guidelines [31], where the femurs are similar to the style on the right in Figure 3. In a new clinic that is starting to use DL-based delineation, there is a learning curve to accommodate a new way of working. To overcome this, the model should also be able to adapt to certain local delineation protocols. While the model is clinically used for autosegmentation, the model could also learn from the clinically edited contours to correspond to the clinic’s way of working. A national and an international contouring database would fully overcome this if every clinic used the same clinical contouring protocol.

When the segmentation model is based on the latest guidelines, it would also challenge experienced specialists to review their current practices. Likewise, the model should be kept up to date. Additionally, the use of the commercial AST studied here could free up time for clinicians to tackle more important delineation tasks (dose painting, intraprostatic boost, etc.), as every contour does not have to be started from scratch. With consistent normal tissue delineation through consensus guidelines and delineation implementation with accurate ASTs across the clinics, the comparability of radiation toxicity studies could increase in the future. The DL-based AST evaluated in this study could provide a basis for improving the accuracy and consistency of the most critical step in radiation delivery to control disease and limit adverse effects related to errors in the delineation of structures in RTP of prostate cancer patients.

## Figures and Tables

**Table 1:** Average similarity scores (DSC, S-DSC and HD95) and average absolute and relative volume differences with one standard deviation (Diff_cm3 and Diff %) between manual contouring (MC) and automated contouring (AC) methods from 30 patients and 45 scans in six clinics by 13 experts.

ROI	DSC	S-DSC (2.5 mm)	HD_95_ (mm)	Diff_cm^3^ (cm^3^)	Diff_% (%)
Bladder	0.93	0.68	3.3	−1.2 ± 12.0	−1.7 ± 8.3
Prostate	0.82	0.38	6.1	−13.5 ± 10.1	−31.6 ± 26.1
Femoral head L	0.68	0.22	25.0	−4.0 ± 32.4	−37.9 ± 60.7
Femoral head R	0.69	0.22	24.7	−3.0 ± 31.2	−30.5 ± 38.7
Lymph nodes *	0.80	0.39	14.7	40.9 ± 86.3	5.2 ± 12.4
Penile bulb	0.51	0.33	7.7	1.2 ± 2.2	−6.8 ± 67.0
Rectum	0.84	0.58	11.4	−4.5 ± 11.4	−9.6 ± 18.7
Seminal vesicles	0.72	0.52	7.1	0.6 ± 6.9	−11.1 ± 31.2

* The lymph nodes were evaluated for five patients and 20 scans while it was performed in one clinic only. ROI: regions of interest, DSC: Dice similarity coefficient, S-DSC: surface Dice similarity coefficient, HD95: 95% Hausdorff distance, L: left, R: right.

**Table 2:** Dice similarity coefficients (DSC) were evaluated between four oncologists for the same five patient data set for manual contouring (MC) and automated and edited contouring (AEC) method. Diff = DSC difference between AEC and MC methods (ROI: regions of interest).

		Oncologist 2			Oncologist 3			Oncologist 4		
	ROI	AEC	MC	Diff	AEC	MC	Diff	AEC	MC	Diff
**Oncologist 1**	Bladder	1.00	0.90	0.10	1.00	0.92	0.08	1.00	0.91	0.09
	Prostate	0.92	0.82	0.10	0.99	0.83	0.17	1.00	0.86	0.14
	Lymph nodes	0.88	0.74	0.14	0.89	0.78	0.12	0.95	0.81	0.14
	Penile bulb	0.82	0.56	0.26	0.87	0.71	0.15	0.80	0.66	0.14
	Rectum	0.97	0.74	0.23	0.97	0.77	0.19	0.98	0.82	0.16
	Seminal vesicles	0.84	0.78	0.06	0.97	0.75	0.22	0.97	0.74	0.24
**Oncologist 2**	Bladder				1.00	0.91	0.08	1.00	0.92	0.08
	Prostate				0.92	0.86	0.06	0.92	0.80	0.12
	Lymph nodes				0.87	0.78	0.10	0.91	0.72	0.19
	Penile bulb				0.82	0.57	0.24	0.90	0.61	0.29
	Rectum				0.99	0.64	0.35	0.99	0.76	0.23
	Seminal vesicles				0.84	0.79	0.05	0.83	0.79	0.04
**Oncologist 3**	Bladder							1.00	0.92	0.08
	Prostate							0.99	0.82	0.18
	Lymph nodes							0.92	0.74	0.18
	Penile bulb							0.80	0.74	0.06
	Rectum							0.99	0.77	0.22
	Seminal vesicles							0.97	0.77	0.20
